# An In Vitro Study of Intaglio Surface, Periphery/Palatal Seal Area, and Primary Bearing Area Adaptation of 3D-Printed Denture Base Manufactured in Various Build Angles

**DOI:** 10.1155/2022/3824894

**Published:** 2022-11-17

**Authors:** Kanyakorn Charoenphol, Chaimongkon Peampring

**Affiliations:** Department of Prosthetic Dentistry, Prince of Songkla University, Hat Yai, Thailand

## Abstract

**Purpose:**

The objective of this research was to compare the adaptation in the overall intaglio surface, peripheral/posterior palatal seal area, and primary bearing area of the 3D-printed complete denture produced in 0, 45, and 90° build angles.

**Materials and Methods:**

A reference edentulous maxillary arch model was scanned to design virtual denture bases with computer-aided manufacturing (CAD) software with standard tessellation language (STL) files as output. Denture bases were fabricated by printing with a digital light processing (DLP) technique and divided into 3 groups according to build angles of 0°, 45°, and 90° (*n* = 10). To assess the adaptation, each STL file of the intaglio denture base was superimposed on the STL file of the reference model using surface-matching software. The adaptation was reported in root mean square error (RMSE) values and statically compared using one-way analyses of variance (ANOVA) and followed by the Turkey's test for multiple comparisons with a significance level of *α* = 0.05.

**Results:**

Overall, intaglio surface adaptation of denture bases printed from three angles had no significant difference in adaption. In the peripheral/posterior palatal seal area, denture bases printed at a 90° build angle showed significantly better adaption than other groups. In the primary bearing area, denture bases printed at 45° and 90° had no significant difference in denture adaptation; however, they exhibited better denture base adaptation than the 0° group significantly.

**Conclusions:**

The build angle has no effect on adaptation in the overall intaglio surface area. The build angle of 90° provided the best adaptation in the peripheral/posterior palatal seal area. The 45° and 90° build angles provided better adaptation than 0° in the primary stress-bearing area.

## 1. Introduction

The effectiveness of a complete denture depends on factors including optimum retention, maximum support, and good stability. The retention is an important factor that allows patients to use dentures with confidence. The design for good retention consists of expanding the maximum area without interfering with the functional limit. Denture bases must be intimately fit to oral tissue especially in the primary stress bearing area and peripheral seal area. The dentures processing is an important factor influencing the adaptation of the denture base to the underlying supporting tissue providing retention, stability, and support for dentures [1]. The conventional denture processing method, compression molding technique, has been used for decades with clinically successful results. However, drawbacks of using this technique are time consuming procedures and multiple clinical and laboratory steps required. Therefore, controlling the quality of a successful complete denture treatment requires expertise [2].

Application of digital technology, such as computer-aided design/computer-aided manufacturing (CAD/CAM) systems in denture fabrication has been recently reported [3]. Digitally assisted denture fabrication provides a major advantage, which is the reduced number of the dental appointments compared to the conventional method. It is suitable for elderly edentulous patients who have underlying diseases or are facing difficulty in coming for dental treatment [4]. There are two types of CAD/CAM-fabricated dentures based on the manufacturing method [5]. Subtractive manufacturing denture is a technique that gives material in the desired shape by milling from prepolymerized polymethylmethacrylate (PMMA) resin block and additive manufacturing denture or 3D-printed denture which parts can be produced from layer-by-layer printing [6]. Historical studies of digitally assisted denture fabrication initially focused on the subtractive method. It was found that both the patient and the operator were satisfied with the dentures from the milling method more than conventional dentures processing [7]. The biocompatibility and mechanical properties of the PMMA discs milled for removable prostheses were acceptable [8]. However, the disadvantage of the subtractive method is the large amounts of waste material it produces. Also, milling procedures can cause damage to the milling burs after being used, causing less accuracy in the milling process [9]. A previous study reported that milled dentures showed better tissue adaptation than the 3D-printed dentures; however, they are both clinically acceptable [9]. Hwang et al. [10] mentioned 3D printing method provides better accuracy of the denture than milling method especially in the case of undercut alveolar ridges. One advantage of the 3D printing procedure compared to the milling method is less time required for manufacturing. Furthermore, less waste material can be produced with the 3D printing method. Recently, research has become interested in creating 3D-printed dentures that can be printed in complex geometry.

The most commonly used 3D-printed technology for a complete denture is digital light processing (DLP), which is a method using a projection of ultraviolet (UV) light to irradiate a single image of the layer across the entire resin at once and processed the workpiece layer-by-layer [11]. The accuracy of DLP printing depends on many factors such as the material used, the thickness of the printed layer, and build angle. A previous study reported that workpieces should be printed at an appropriate angle to minimize errors [12]. Also, the angle of the workpiece made with the platform during printing will affect the accuracy [13]. It was found that the angle that gave the best accuracy for 3D-printed temporary crowns was 135° [14]. However, a denture base has different geometry from a temporary crown; therefore, different build angles could affect the accuracy of the denture base. There has been lack of study involving the optimum build angle to provide as good tissue adaptation of the 3D-printed denture base. Thus, the purpose of this research was to compare the adaptation in the overall intaglio surface, peripheral/posterior palatal seal area, and primary bearing area of the complete denture base produced by DLP printing technology printed at different building angles. The null hypothesis was that the adaptation of the denture bases manufactured by DLP printing at different build angles in the overall intaglio surface, peripheral/posterior palatal seal area, and primary bearing area was no different, statistically.

## 2. Materials and Methods

### 2.1. Master reference Model

**Figure 1:**
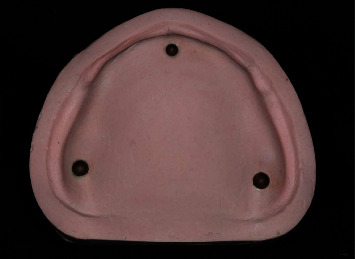
An edentulous maxillary reference model with 3 referent balls.

An edentulous maxillary reference model was fabricated with residual ridge morphology resembling the American College of Prosthodontists type A classification [15]. Three metal spheres were placed over the master model of which two spheres were located on the crest of the ridge over each tuberosity, and one sphere was placed at the center of anterior ridge. They were landmarks for precisely superimposition of a virtual reference model and intaglio surface of the denture base to ensure that the measurements were made at the same location (Figure 1). The reference model was digitized using an extraoral scanner (E4 scanner, 3 Shape Dental System) to create a virtual maxillary model in CAD software (3 Shape Software, 3 Shape Dental System). A scanned file was exported as a standard tessellation language (STL) file. A virtual denture base was designed with 2 mm thickness by using the same CAD software for fabricating a virtual model and also saved as an STL file.

### 2.2. Study Specimens and Data Scanning

**Figure 2:**
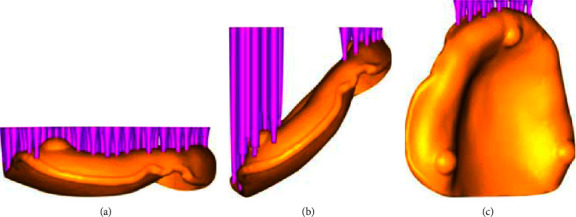
Three different build angles used in this study. (a) 0°. (b) 45°. (c) 90°.

The STL files of the designed denture base were exported to 3D printing software (Asiga Composer, Asiga, Alexandria, NSW, Australia) for printing preparation. Denture bases were fabricated with a 3D printer (Asiga Max, Asiga, Alexandra, NSW, Australia), which is a digital light processing system. The printed layer thickness was set at 100 *μ*m and the wavelength of the light source was 385 nm. The denture bases were printed by placing the workpiece at a different angle to the platform while printing with photopolymerized resin material (Optiprint Gingiva, Dentona, Dortmund, Germany) with the compositions of aliphatic urethane methacrylate, tetrahydrofurfuryl methacrylate difunctional methacrylate, and phosphine oxide. Thirty denture bases were printed and divided into 3 groups (*n* = 10): group 1 was with an angle of 0°, group 2 with 45°, and group 3 built with an angle of 90° relative to the printing platform (Figure 2). Assuming a large effect size and type I and type II error probabilities of 0.05 and 0.95, respectively, a sample size of approximately ten is required to have 80% power to detect differences among groups [16]. Support structures were only located at the polishing surface because the intaglio surface of the denture base was to be examined. After printing, all denture bases were postprocessed following the manufacturer's recommendation by cleaning twice with 99% isopropyl alcohol for 3 minutes followed by postpolymerized for 30 minutes by a UV polymerization unit (Asiga Flush, Asiga, Alexandria, NSW, Australia). After that, the supporting structures that attached to the polishing surface were removed by gentle grinding with a carbide bur and rotating handpiece.

### 2.3. Measurement of Adaptations

**Figure 3:**
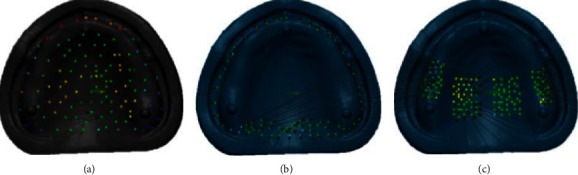
The adaptation evaluation performed in three areas. (a) Overall intaglio surface with 105 measuring points. (b) Peripheral/posterior palatal seal area with 72 measuring points. (c) Primary bearing area with 140 measuring points.

For adaptation measurement, the intaglio surfaces of printed denture bases were scanned and saved in an STL file using the previously mentioned extraoral scanner. Each STL file of the intaglio surface of the denture was superimposed with the STL file of the reference model using first an initial alignment and then best-fit alignment in a 3D surface-matching software (Geomagic Control *X*, 3D Systems, Rockhill, SC, USA). The adaptation of this study was obtained by RMSE by dividing the sum of all the absolute values of the deviation, which are the distance between point clouds of the reference model and the intaglio surface of denture bases. The RMSE value, which was close to zero, meant the good adaptation of the denture base. The adaptation evaluation was performed in three areas as follows: (1) the overall intaglio surface with 105 measuring points, (2) the peripheral/posterior palatal seal area with 72 measuring points, and (3) the primary bearing area with 140 measuring points, as shown in Figure 3. Subsequently, a color map was created for qualitative expression. The nominal deviation was set at +50 *μ*m and the maximum critical deviation was set at +300 *μ*m.

### 2.4. Statistical Analysis

Data were statistically analyzed using SPSS 24.0 for Windows (SPSS, Chicago, IL, USA). The Shapiro–Wilk test found that the data were normally distributed and the homogeneity of variance was satisfied according to the Levene's test. Therefore, the averages of the RMSE values at 0°, 45°, and 90° were statically compared using one-way analyses of variance (ANOVA) and followed by the Turkey's test for multiple comparisons with significance level of *α* = 0.05.

## 3. Results

Average RMSE values and standard deviations for three evaluation areas were listed in Table 1. The one-way ANOVA test indicated that there was no significant different in the overall intaglio surface adaptation of denture bases printed from three various angles (*P*=0.497). Considering the peripheral/posterior palatal seal area, denture bases printed at a 90° build angle showed the least deviation from the master model meaning that they demonstrated the best adaptation compared to denture bases printed at 45° and 0° build angles. Furthermore, the average RMSE adaptation of the denture base on the primary bearing area showed a significant difference between the 0° group and the other groups. The angles for constructing denture bases at 90° and 45° demonstrated no significant difference in denture adaptation; however, they exhibited better denture base adaptation than the 0° group significantly.

**Figure 4:**
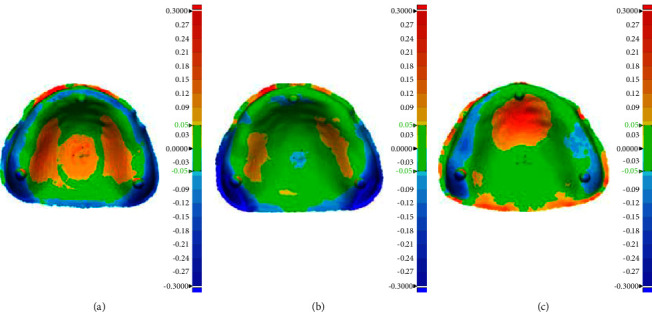
The color mapping demonstrated deviation from reference model of denture bases printed with different build angle. (a) 0°. (b) 45°. (c) 90°.

The color mapping that showed up in yellow or red color demonstrated positive deviation which meant that there were spaces between the denture base and the reference model. On the contrary, color mapping represented in cyan or blue color demonstrated the negative deviation, which meant that there was compression of the denture base against the reference model. The green color can be interpreted as the ideal intimacy of the denture base and reference model and the RMSE value would be approximately 50 *μ*m. In the group of 90° build angle, color mapping illustrated mostly in green color, especially around the periphery and posterior palatal, while the groups of 0° and 45° build angle demonstrated different color mapping results, as shown in Figure 4.

## 4. Discussion

The results in this study indicated that there was no significant difference in the overall intaglio surface adaptation of denture bases printed from three various angles while the adaptation at the peripheral/posterior palatal seal area and primary bearing area showed statistically significant differences. In the peripheral/posterior palatal seal area, the 90° build angle of the printed denture base provides the best adaptation, followed by 45° and 0°, respectively. In the primary stress-bearing area, the 45° and 90° building angles provided better adaptation than 0°. Therefore, the null hypothesis is partially rejected.

In the edentulous jaws, mucosal tissues covering the maxilla and mandible provide support and retention for a complete denture. There are some mucosal areas that can bear pressure and some are unable to be loaded due to the different types of soft tissues and bony structure. The primary stress-bearing area generally possesses thick keratinized mucosa and hard cortical bone, which is subjected to less resorption during function. The intimately adapted denture bases at primary stress-bearing area will help in good retention and stability [17]. In the case of denture bases are firmly attached to peripheral/posterior palatal area, this will create an almost vacuum between the denture and the underlying tissue and the air cannot penetrate under the denture bases. This will cause the external atmospheric pressure applied to the polished surface to be greater than atmospheric pressure acting on the tissue side, resulting in good retention of the denture base [1]. Therefore, this study focused not only on overall intaglio surface adaptation evaluation but also on denture base adaptation in the primary stress-bearing area and peripheral/posterior palatal seal area.

Several techniques have been applied to assess the degree and location of dimension change that occurs during denture processing. These have included sophisticated 2-dimensional and 3-dimensional measurements. Recently, extraoral scanners combined with surface-matching software have gained popularity as a method for measuring denture base adaptation [18]. The adaptation of this study was obtained by RMSE by dividing the sum of all the absolute values of the deviations, which are the distance between the reference model's point clouds and the surface of the scanned model. Adaptation analysis using these techniques has previously been described in [18–20].

The results from this study indicated that different build angles affected denture base adaptation in the peripheral/posterior palatal seal and primary bearing area, which could be explained by 2 reasons. First, during the 3D printing procedure, the designed structure is built in layer-by-layer, which creates a staircase effect which is caused by the offset between layers in oblique and curve area creating noticeable steps on the surfaces. More steps appear when the structure is printed on large curved surfaces and the distance between two consecutive layers increases. The peripheral/posterior palatal seal area and primary bearing area of denture bases appear to be in a large oblique and curve area in which the staircase effect could influence the denture adaptation. The staircase effect can be reduced by optimizing the build angle [21]. The workpiece should be placed at an angle that allows for a gradual change between two consecutive layers of printing. In this study, the staircase effect was obviously seen when printing direction was a 0° while the 45° and 90° build angles showed less staircase effect. As a result, the denture base printed with 0° build angle exhibited worse denture adaptation in the palatal seal and the peripheral seal area and primary bearing area compared with the 45° and 90° groups.

Second, each printed object requires supports during printing according to the principle of 3D printing method. Each successive layer is printed on top of the previous layer, which then creates the 3D structure causes the material with a more support area to expose more UV light and shrink towards the supporting structure. This was in accordance with a previous study [14] in which the printed temporary crowns had better accuracy when less amount of support was used. In this study, the lowest numbers of supports was 90° build angle group followed by 45° group. The 0° build angle group exhibited large numbers of supports due to large horizontal area while being printed in each layer causing more tendency of printed structure to be distorted. As a result, the 0° group showed poor adaptation of denture bases, especially in the posterior palatal seal and the peripheral seal area and primary bearing area.

The finding in this study contradicts a previous study that investigated the influence of build angle on the accuracy of printed objects using the stereolithographic (SLA) technique [22]. The result showed that denture bases printed at an a build angle of 45° had better accuracy than 90° and 0°, respectively. The 0° build angle had the lowest adaptation. In this study, the results appeared to be different from the previously mentioned study because a different technology of printing (DLP) was used. However, the group of 0° build angle had the lowest accuracy, which is in consistent with the previously mentioned study because at 0° was found to have the most noticeable staircase effects. The layer thickness of printing was set at 100 um in the current study as it was recommended by a previous study, which found that 100 *μ*m layer thicknesses provided better accuracy of printed structure [23].

When the denture base was printed at a 0° build angle, it occupied the most space on the platform, thus only a small number of complete dentures can be printed and a time and it required approximately 45 minutes of printing time. A denture base printed at 45° build angle used less space in platform than 0° but it more printing time which was approximately 1 hour. The denture base printed at a 90° build angle used the least amount of space on the platform enabling multiple complete dentures to be printed at one time and it required approximately 1 hour and 20 minutes to print. Moreover, the 90° build angle denture has the lowest number of supports therefore saving materials and time for removing supports than others. As a result, the denture surface became smoother than at other angles.

One limitation of this study was that the intaglio surface adaptation of the denture base was determined in an extraoral condition. The oral mucosa has dynamic characteristics of compress soft tissue which is not simulated in this study. It was found that the oral tissue was able to be compressed to 375–500 *μ*m when dentures were inserted [24], which was greater than the critical deviation of this study (300 *μ*m). Therefore, the 3D-printed denture bases fabricated from 3 different build angles possessed the clinical acceptable adaptation. This study was performed on specific ridge morphology; therefore, it cannot be extrapolated to other edentulous ridge morphologies. Other factors such as saliva immersion, in this study and needed to be investigated in the future.

## 5. Conclusions

Within the limitations of this in vitro study, the following conclusions could be drawn. The different build angles while printing have no effect on adaptation in the overall intaglio surface area of denture bases. The build angle of 90° provided the best adaptation in the peripheral and posterior palatal seal areas. On the other hand, the 45° and 90° build angles, which provide better adaptation than 0° in primary stress-bearing area. However, the adaptation of 3D-printed denture bases in this study was at a clinically acceptable level.

## Figures and Tables

**Table 1:** Descriptive statistics showing RMSE values (the mean ± standard deviation in mm) of three different area of measurements.

Groups	0°	45°	90°
Overall surface area	0.1209 ± 0.0033^*a*^	0.1265 ± 0.0036^*a*^	0.1219 ± 0.0037^*a*^
Peripheral and posterior palatal seal areas	0.2245 ± 0.0086^*A*^	0.1966 ± 0.0060^*A*^	0.1635 ± 0.0040^*B*^
Primary bearing area	0.0618 ± 0.0018^*α*^	0.0408 ± 0.0018^*β*^	0.0498 ± 0.0031^*β*^

Same letters in each row indicate statistically no significant difference (*α* = 0.05).

## Data Availability

The data that support the findings of this study are available from the corresponding author upon request.
